# A Fuzzy Multiple Criteria Decision Making Approach with a Complete User Friendly Computer Implementation

**DOI:** 10.3390/e23020203

**Published:** 2021-02-07

**Authors:** Ludmila Dymova, Krzysztof Kaczmarek, Pavel Sevastjanov, Joanna Kulawik

**Affiliations:** Department of Computer Science, Czestochowa University of Technology, Dabrowskiego 73, 42-201 Czestochowa, Poland; dymowa@icis.pcz.pl (L.D.); krzysztof.kaczmarek@icis.pcz.pl (K.K.); joanna.kulawik@icis.pcz.pl (J.K.)

**Keywords:** hierarchical, fuzzy, MCDM, implementation

## Abstract

The paper presents the generalization of the almost forty years of experience in the field of setting and solving the multiple criteria decision-making (MCDM) problems in various branches of a human activity under different types of uncertainties that inevitably accompany such problems. Based only on the pragmatic intentions, the authors avoid the detailed descriptions of the known methods for the decision-making, while instead focusing on the most frequently used mathematical tools and methodologies in the decision-making practice. Therefore, the paper may be classified as a special kind of illustrative review of the mathematical tools that are focused on applications and are the most used in the solutions of MCDM problems. As an illustrative example, a complete user-friendly computer implementation of such tools and methodology is presented with application to the simple “buying a cat” problem, which, however, possesses all the attributes of the hierarchical fuzzy MCDM task.

## 1. Introduction

Uncertainty is an inherent property of the decision-making problems. Even when we deal with the two local criteria, an uncertainty is, for the most part, of a subjective nature concerned with the local criteria relative to importance assessment. There are different types of uncertainty that are taken into account when solving the MCDM tasks dependent on the problem considered. Among them, the following types of uncertainty and uncertainty focused theories in application to the MCDM should be dedicated: interval uncertainty [1], ordinal fuzzy sets theory [2], interval-valued fuzzy sets theory [3], type 2 fuzzy sets [4] (see the recent comprehensive review in [5] and the last applications in [6]), intuitionistic fuzzy sets [7], interval-valued fuzzy sets [8], hesitant fuzzy sets [9], Dempster–Shafer theory of evidence [10], evidential reasoning [11], rule-base evidential reasoning [12], rough sets theory [13] and soft set theory [14].

There are a number of papers in the literature devoted to the solution of MCDM problems—the somewhat exotic new theories such as the L-fuzzy sets, the neutrosophic fuzzy sets, the Pythagorean fuzzy sets, the cubic fuzzy sets and so on. Of course, they can be considered as the innovative theories. Nevertheless, although a lot of theorems were proven, we did not find in the literature any real-world (non-artificial) applications of them. A similar situation was observed 20–30 years ago, when many new innovative (but exotic) theories such as relativistic fuzzy sets and quantum fuzzy sets were discussed. Who remembers them now? Of course, this does not mean that such new innovative exotic theories should be discarded; on the contrary, they should be thoroughly investigated not only from the point of view of their mathematical validity (as it is mostly done now), but also from the point of view of their practical applicability. Nevertheless, they are not yet mature enough to be recognized as the reliable approaches able to process an important uncertain information. Therefore here we will not analyze such theories since this paper is focused strictly on the approaches that already proved their applicability. On the other hand, we can say that the applicability-focused analysis of new innovative theories is the main direction of our future studies.

Of course, the above short review is not comprehensive and demonstrates only a variety of approaches used in MCDM to take into account different kinds of uncertainty.

There are many problems caused by the specificity of different types of uncertainty, e.g., the choice of the proper operational laws in intuitionistic fuzzy sets theory, the comparison of uncertain values and so on, but the common problem is the choice of an appropriate convolution mode. The problem of the determination of local criterion weights is most often solved using a pairwise comparison matrix, although the methods based on the estimation of the local criteria reliability are applied as well. The hierarchical MCDM problems are usually solved using the well-known analytic hierarchy process (AHP) [15,16], which unfortunately possesses too many disadvantages. We have experience in the use of different types of uncertainty in MCDM, and our contributions to the solution of these problems are partially presented in [17,18,19,20,21,22,23,24,25,26,27,28,29,30,31,32,33,34,35,36,37,38,39,40,41,42,43].

Nevertheless, our experience, accumulated during almost forty years practice of development and implementation of the MCDM support systems in Russia, Belarus and Poland [44,45], allows us to conclude that in practice there are relatively few situations when it is necessary to take into account these complex types of uncertainty. Often this does not mean that they do not really exist objectively, but the decision makers and the experts involved do not always have the necessary knowledge and skills to identify and formalize them.

Therefore, we should recognize that the vast majority of publications of an applied nature in the field of MCDM are based only on the use of ordinary fuzzy sets. Additionally, it is important that according to our observations and our own experience, objectively there is a huge amount of research in the field of fuzzy MCDM which cannot be published in order to keep trade secrets.

Therefore, the aim of this paper is to present the selected set of mathematical tools and methodologies that proved their effectiveness and reliability in the solution of MCDM problems during our (and not only our) many years of practice. Thus, we want to share our experience with those who have encountered MCDM problems and should choose the appropriate mathematical tools for their solutions. As a consequence, the paper can be classified as an unusual practice-oriented review of such selected tools. Of course, a considerable number of them are already presented in the literature, but here we represent them while focusing on their ability to build jointly complete systems for MCDM support.

It is important that such practical approaches do not prevent the presentation of some relevant new results and methods.

Now we are able to present a selected set of mathematical tools that can be used to formulate and solve most of the real-world MCDM problems. Some of them, e.g., pairwise comparison matrices used to get local criterion weights, are well known. The other tools we have developed, such as the generalization of convolution modes, are not so popular although could to be very useful in practice providing consensus solutions. We can say the same about our approach to the aggregation of hierarchical structures of local criteria. In this paper, we present these selected tools using the seemingly toy example of “cat buying” problem, which, however, turns to be a simple, but complete fuzzy hierarchical MCDM problem. The solution procedure will be presented by the screenshots of the friendly computer system we have developed for the solution of such MCDM problems. It should be emphasized that the choice of the “cat buying” example was due to the desire to use as an illustration some compromise between academic rigor and the specificity of the real-world problem. In any case, we hope that this example will be understandable to all readers. Of course the presented software may be successfully used for the solution of a relatively wide range of MCDM problems, not only those in pet shops.

We hope this will convince the practitioners that creating valuable and complete software for the hierarchical fuzzy MCDM support is not so difficult a problem.

Concerning the originality of this paper, we can say that it first presents a new special kind of illustrative review of the selected mathematical tools that are focused on the applications and most used in the solution of the MCDM problems, and this approach to reviews can be treated as an innovative one. The main contribution of this work is the justified (also based on the authors’ 40 years of experience) selection of mathematical tools able to build in their synthesis reliable systems that can be used for the solution of fuzzy hierarchical MCDM problems in a broad range of fields.

The rest of the paper is organized as follows. In Section 2, the selected set of mathematical tools that have proven their effectiveness for solving MCDM problems is presented with the remarks concerned with their usage. Section 3 illustrates step by step the software we developed using the screenshots of the solution of a hierarchical, fuzzy MCDM “cat buying” problem. Section 4 concludes the paper with some remarks.

## 2. The Basic Concepts and Mathematical Tools Used

**Figure 1:**
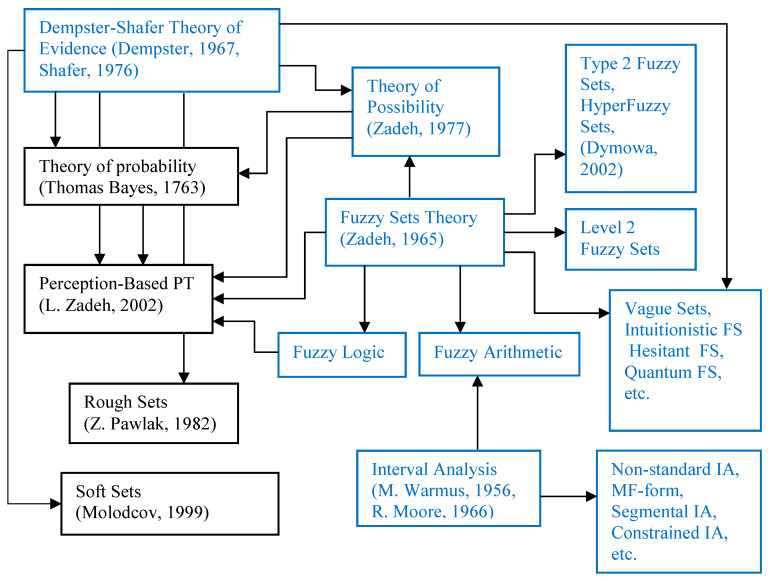
The proposed linked structure of modern uncertainty theories (under discussion).

Based on our knowledge and therefore in a somewhat debatable way, the genesis and actual structure of uncertainty theories with their links may be (not completely) presented as in Figure 1.

We have contributed to the development of some of them (marked by the blue color in Figure 1) in the context of the MCDM problems. It is seen that the more general Dempster–Shafer theory of evidence (DST) generates particularly many (asymptotic) cases of the second order theories. We have successfully used this generalizing ability of DST for the generalization of Atanassov’s intuitionistic fuzzy sets [28], interval-valued intuitionistic fuzzy sets [21], hesitant fuzzy sets [23] and fuzzy logic [22] in the framework of DST. This allowed us to avoid the limitation and shortcomings of the more frequently used second order theories (intuitionistic, hesitant, etc.) and enhance the performance of the methods developed for the solution of the MCDM problems. Hence, we can state that we are familiar enough with the modern uncertainty theories, and contributed to their development and applications in the field of MCDM.

Nevertheless, here we will deliberately limit ourselves to only considering the classical fuzzy type of uncertainty as the most used in practice.

**Figure 2:**
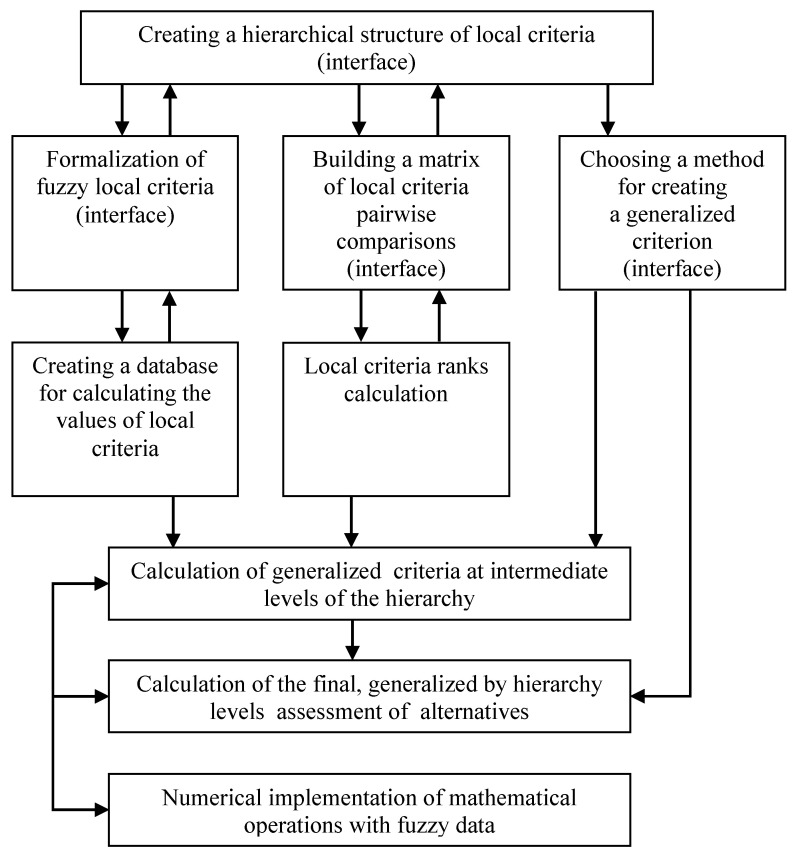
The enlarged structure of the proposed methodology and an implementation of MCDM.

Therefore, here we will present only our approach to the solution of the MCDM problems, which provided valuable results in solving the different real-world problems we met in our practice. To make reading the paper easier and for a better understanding of our approach, its structure reflecting the enlarged implementation is presented in Figure 2. The components of this structure are described in detail in the corresponding subsections below.

The above structure (sometimes somewhat extended) we have used to solve MCDM problems, e.g., in budgeting [37,43], in steel selection [36], in logistics [19,44], in stock [35,36,38], in stock level 2 quotes [30], currency trading systems [22] and in type 2 diabetes diagnosis [25,32] (here we cite only the papers published in the most reputed journals). Hence, we can say that the proposed approach performs well in the broad range of applications in different fields from technology and logistics to finance and medicine.

### 2.1. The Local Criteria Mathematical Formalization

When a set of local criteria and their hierarchical structure are established, a more complex stage of their mathematical formalization begins. Probably the first approach to formalize local criteria free of the limitations of classical utility theory, e.g., such that a utility must be a non-decreasing function, was the concept of desirability function introduced by E.C.J. Harrington [46] in 1965. This concept was primarily focused on the solution of multiple criteria optimization problems. The desirability function may be defined as follows. For each value of optimized variable, a desirability function assigns the value lying in the interval [0,1] to the admissible values of this variable—zero for a completely undesirable value of the variable and 1 representing a completely desirable or ideal value. The specific type of desirability functions is set out by the decision makers based on their subjective perceptions. By aggregation of partial desirability functions (usually using the geometric mean operator) taking into account their weights, a global criterion for the quality of the process is constructed, the maximization of which delivers the optimum. This approach is currently successfully used for the optimization in different fields (see, e.g., the overview in [47]), especially in planning experiments when searching for the optimal conditions. It has been used most successfully used in optimizing the processes of chemical technology, material processing, metallurgy and a number of other industries.

Almost simultaneously, in 1965 year, L.A. Zadeh introduced the cornerstone concept of his new “fuzzy sets theory”, i.e., the definition of a membership function [48]. In the framework of decision-making and optimization problems, the context and even formal definitions of desirability and membership functions were equivalent until 1970 when the seminal paper by Bellman and Zadeh [49] devoted to decision-making in a fuzzy environment was published; the fuzzy set theory and its conceptual and mathematical formalism were primarily applied to deal with mathematical objects of a linguistic nature. Since then, a growing number of studies in the field of fuzzy and extended fuzzy optimization and decision-making problem solutions were released. Meanwhile, Harrington’s method was not supported by attractive new concepts and constructive mathematical tools, and therefore it was not so widely developed. Currently it is used, rather, as a useful practical technique for optimal planning in industrial experiments when searching for the optimal conditions.

On the other hand, the main attraction of the fuzzy sets theory is that it allows a decision maker to use more of the different types uncertain information in the problem formulation and its solution. Therefore, hereinafter we will use the membership functions to formalize fuzzy local criteria, as these functions are based on the more fruitful semantics of the fuzzy sets theory rather than the desirability functions.

The formalization of a membership function in practice usually is not a difficult problem. In many cases, in the production quality management the technological instructions may serve as a direct method to build membership functions of the product quality criteria. For example, according to such an instruction, the value of the product’s quality parameter should be in the interval of admissible values and this interval may be naturally assumed to be the support of the trapezoidal fuzzy membership function. On the other hand, the value of quality parameter should be, if possible, in the interval of the best parameter values included in the interval of the admissible values.

**Figure 3:**
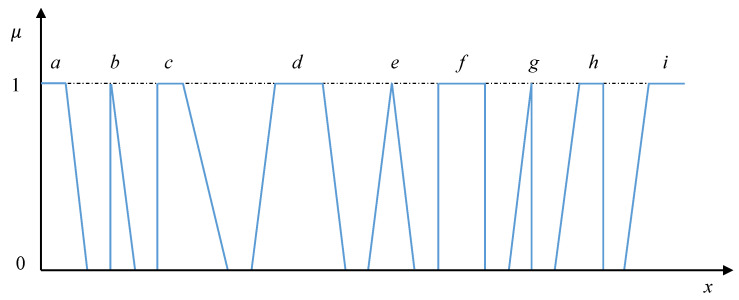
The most used types of membership functions representing local criteria.

Obviously, the interval of the best parameters values may serve as the top of the trapezoidal fuzzy membership function. In a similar way, the triangular, rectangular, right and left trapezoidal membership functions representing local criteria may be formalized. Hence, the most used in the MCDM shapes of membership functions are presented in Figure 3.

In practice, we can meet the extremely competent experts or the decision makers who can draw more complex membership functions (of course convex ones and with one maximum equal to 1). However, they often cannot clearly explain why the function should look so difficult. It is interesting that the typical answer to such questions is something like that: “After working in my workplace for 15–20 years you would draw the same thing yourself”. In general, this is not surprising, since the long term experience of experts and their developed intuition, accumulated largely subconsciously—due to the properties of human thinking—are more easily transmitted graphically than verbally. This is a remarkable feature of the fuzzy set theory, i.e., an ability to process the information presented even on the intuition based or graphical levels.

### 2.2. The Calculation of Local Criteria Relative Importance

We can say that if a decision maker told you that in the MCDM problem at hand the local criteria have a same importance, this rather means that he/she is not competent enough. In our practice, we met such equally important local criteria only in the textbooks (also in our books and some our papers for purely methodological purposes). Meanwhile, the choice of relative importance (weights) of local criteria is usually a more difficult task for a decision maker than the local criteria formulation. However, if he/she has problems with the ranking of to say 10 criteria, he/she usually can easily compare the pairs of them. It also turns out that decision makers are much more willing to give verbal assessments of relative importance in pairs of compared criteria while avoiding numerical estimates. This is an inherent property of a human thinking. Such observations served for T. Saaty [15] as a methodological basis for the introduction of the so-called linguistic pairwise comparison matrices, which in turn are a cornerstone of the famous analytic hierarchy process (AHP). We will consider the AHP as a whole in Section 2.4; here we focus only on the practical features of using the linguistic pairwise comparison matrices to calculate the weights of local criteria.

The fist step is the linguistic pairwise comparison matrix building.

The best practice is to obtain primarily from a decision maker the verbal assessments of relative importance of local criteria pairs. The reason behind this is as follows.

It is established that when we ask a group of experts to scale or rank some known objects, they usually provide practically coinciding verbal estimations. No wonder, since these experts learned from the same textbooks and articles used in the field. However, if we can get them to provide numerical estimates (which is usually not easy to do), there will be no consensus in the resulting assessments [50].

It has long been established that generally people prefer words to numbers [51] so that the use of verbal equivalents is considered to be beneficial for the recipients of expert information who do “not feel confident in handling numbers and react negatively to mathematical formulae” (p. 447) [52]. Therefore, people are willing to give verbal estimates, avoiding if possible numerical ones, in which they are usually not very sure. This empirically justified fact is well known in applied psychology and often used in practice, e.g., in marketing studies [50].

This is easy to explain. The fact is that “At first there was a Word” (The Holy Gospel of Jesus Christ). Numbers were invented much later and during the last 4–5 millennia people simply did not have time to really learn how to handle them, and apparently that is why they are trying hard to shift this work to their computers. Therefore, currently we prefer to think using words, not numbers. Regrettably, the human ability to do this in the context of our problem of verbal ranking local criteria is strongly restricted by the known empirical law of psychology, according to which a person can normally distinguish no more than seven plus or minus two classes or grades on some feature scale. If the number of grades is greater, the adjacent grades start to merge and cannot be clustered confidently (see [53,54]). Therefore, in all natural languages, the number of verbal estimates of relative importance does not exceed nine. They are presented in Table 1. Of course, they can be represented by another word combinations, but their sense and number remain the same.

The conventional values on the right hand side of Table 1 are assigned to the linguistic estimates to make possible the calculations that provide the real-valued weights of local criteria.

Consider an example of filling a pairwise comparison matrix for the criteria X, Y and Z. The linguistic estimations of relative importance are as follows:

- X is a bit stronger than Y;
- Y is somewhat stronger than Z;
- X is significantly stronger than Z.

The pairwise comparison matrix will therefore look like that in Table 2.

Let the denote a component of a pairwise comparison matrix as $A_{ij}$ and the importance (weight) of *i*th local criterion as $w_{i}$. Then for i,j=1…n.

(1) $$A_{ij}=\frac{w_{i}}{w_{j}}$$

Hence, the problem is to calculate the local criterion weights based on the corresponding pairwise comparison matrices.

There are many different approaches proposed in the literature to obtain these real-valued weights. In [55], their approximate classification was presented as follows: the eigenvector method, the least squares method (LSM), the logarithmic least squares method (LLSM), the geometric row means method (GRM), the weighted least squares method (WLSM) and a category of methods that involve only arithmetic operations: the row means of normalized columns approach [15], the normalized row sum and the inverted column sum methods [56].

The broad studies of these methods advantages and shortcomings were presented in the literature for the case of real-valued pairwise comparison matrices [57] and for the fuzzy-valued matrices as well [55,58]. Nevertheless, because really we have only an approximate equality in expression (1), the best criterion of pairwise comparison accuracy can be formulated as follows [57]. Let $\left(w_{1},w_{2},\ldots ,w_{n}\right)$ be the vector of unknown local criterion weights. In real situations, the elements of the pairwise comparison matrix generally are not accurate due to the fact that they reflect the subjective opinions of experts. Under these conditions, the vector of approximate values of local criterion weights $w_{i}$, *i* = 1 to *n* can be obtained as a vector minimizing the functional $F=\sum_{i=1}^{n}\left(\sum_{j=1}^{n}{\left(A_{ij}-\frac{w_{i}}{w_{j}}\right)}^{2}\right)$. Therefore the desired values of $w_{1},w_{2},\ldots ,w_{n}$ were found from the solution of the following optimization task:

(2) $$F=\sum_{i=1}^{n}\sum_{j=1}^{n}{\left(A_{ij}w_{j}-w_{i}\right)}^{2}\;\to \;min;\;\sum_{i=1}^{n}w_{i}=1.$$

Based on a large number of experimental data, it was shown in [59] that the method (2) (LSM) seems to be the best one as it provides the minimal final (optimal) values of *F*. Nevertheless, sometimes the simplest row means of normalized columns Saaty’s method [15]

(3) $$w_{i}^{{}^{\prime }}=\sqrt[{n}]{\prod_{j=1}^{n}A_{ij}},\;w_{i}=\frac{w_{i}^{{}^{\prime }}}{\sum_{i=1}^{n}w_{i}^{{}^{\prime }}}$$

provide the results acceptable from the practical viewpoint [59].

This opinion concerned with the accuracy of the approximate approach (3) seems to be an unduly optimistic one. Therefore, to get a numerical estimation of the inaccuracy of the simplified approach (3) we have used the known example of the private house evaluation from the book [60]. The corresponding pairwise comparison matrix is presented in Table 3.

An informative indicator of the reliability of determining ranks is the so-called inconsistency index (I) of the matrix of pairwise comparisons *A*, which gives information about the degree of violation of the numerical (cardinal $A_{ij}=w_{i}/w_{j}$) and transitive (ordinal) consistency of the expert estimates. If there is poor consistency, we recommend searching for additional information and reviewing the data used to build the scale. The inconsistency index for each matrix is calculated based on an estimate of the maximum eigenvalue of the matrix $e_{max}$.

It can be approximated as follows: first, each column of judgments is summed; then the sum of the first column is multiplied by the value of the first component of the normalized priority vector; the sum of the second column is multiplied by the second component; and *so on*. This results in the value $e_{max}$. The inconsistency index I is calculated using the expression $I=\left(e_{max}-n\right)/\left(n-1\right)$, where *n* is the number of elements to compare. For a reciprocal matrix, $e_{max}\geq n$ always. The lower the inconsistency index is, the better the consistency of expert opinions. Based on the inconsistency index I, an indicator of the inconsistency ratio is calculated: IR = I/C, where C is the value of the random inconsistency of the matrix of the same order.

The average values of the inconsistency C for random matrices of different orders obtained by random selection of quantitative judgments from the scale 1/9, 1/8, 1/7, ..., 1, 2, ..., 9 and the formation of an inverse-symmetric matrix, are shown in the Table 4:

In [60], it was found that in order to consider the expert judgments consistent, the value of the IR must be less than 10%. In some cases, an IR value of up to 20% can be considered acceptable for practice with large dimensions of matrices. If the IR goes beyond these limits, then experts need to review the task and check their judgments.

In relatively large matrices, starting from 7 to 9 elements, it is often difficult to achieve a high level of consistency. However, the level of consistency should be consistent with the risk associated with working with inconsistent results. For example, when comparing the effects of drugs on the body, due to the special responsibility of the task, it is necessary to have a very high level of consistency.

The Table 5 shows the results of calculating the ranks, I and IR, for the example of ranking criteria for evaluating a house (Table 3) obtained using the two compared methods.

As follows from the analysis of the results, the values of ranks obtained using the compared methods may differ considerably. At the same time, the consistency of estimates when using the optimization method (2) is only somewhat better than that obtained using the approximate method (3). This indicates the advantages of the method based on solving the optimization problem (2) in comparison with the traditional approximate approach. Meanwhile, although the ranks of local criteria obtained using the compared methods significantly differ (see Table 5), they provide the same qualitative rankings of local criteria. Hence, we can say that the approximate solution (3) may be used in practice, however, with great caution.

In many practically important cases, for example, when comparing the degrees of significance of individual characteristics in the overall assessment of health status and ranking the criteria of the quality of investment projects, often the groups of experts participate in the construction of pairwise comparison matrices. In such situations, the components of the source pairwise comparison matrices will contain some sets of numbers. The simplest way to solve the problems that arise in this case—operating with the average values of these sets of numbers—obviously leads to a significant loss of information. Therefore, depending on the quantity and quality of the source data, they can be aggregated in the form of crisp or fuzzy intervals for each component of the pairwise comparison matrices. The use of frequency distributions is usually impractical due to the lack of data necessary for their construction, and the inability to perform arithmetic operations directly with them. It is clear that the results of calculating the relative importance (weights) based on the matrix of pairwise comparison with components being intervals or fuzzy numbers will be intervals or fuzzy numbers as well.

To solve this problem, we consider a method based on the fuzzy extension of the approximate method (3). The use of an approximate method as the basis in this situation is justified by the very fuzzy, approximate nature of the source data and by the natural requirement of matching to some extent the accuracy and mathematical complexity of the method with the degree of uncertainty of the source data.

In practice, the method is reduced to replacing a real-valued component in the expressions (3) with their interval or fuzzy representations. The fuzzy extension of the expressions (3) requires performing operations of fuzzy interval multiplication, addition and division, and raising the fuzzy value to the power of an arbitrary fuzzy number. Since these operations are well presented in the numerous textbooks, here we present only the power operation with both operands being fuzzy values, which is seldom shown in manuals, but often happens to be very useful in the solutions of MCDM problems.

A generalized method for raising a fuzzy value to a fuzzy degree is based on the general methodology for transforming fuzzy numbers to the sets of intervals on α-cuts and interval extension of functions.

Let μ and *a* be fuzzy intervals; then in accordance with the rules of partitioning into α-cuts and interval extension of functions, raising a fuzzy interval μ to a fuzzy degree *a* can be represented as $\mu^{a}={⋃}_{\alpha }\mu_{\alpha }^{a_{\alpha }}$, where $\mu_{\alpha }=\left[{\underline{\mu }}_{\alpha },{\bar{\mu }}_{\alpha }\right]$, $a_{\alpha }=\left[{\underline{a}}_{\alpha },{\bar{a}}_{\alpha }\right]$ are crisp intervals corresponding to α-cuts.

In accordance with the general methodology for constructing interval arithmetic, the expression for a crisp interval that is the result of rising $\mu_{\alpha }$ to the degree $a_{\alpha }$ is represented as follows:

(4) $$\mu_{\alpha }^{a_{\alpha }}=\left[min\left\{{\underline{\mu }}_{\alpha }^{{\underline{a}}_{\alpha }},{\underline{\mu }}_{\alpha }^{{\bar{a}}_{\alpha }},{\bar{\mu }}_{\alpha }^{{\underline{a}}_{\alpha }},{\bar{\mu }}_{\alpha }^{{\bar{a}}_{\alpha }}\right\},max\left\{{\underline{\mu }}_{\alpha }^{{\underline{a}}_{\alpha }},{\underline{\mu }}_{\alpha }^{{\bar{a}}_{\alpha }},{\bar{\mu }}_{\alpha }^{{\underline{a}}_{\alpha }},{\bar{\mu }}_{\alpha }^{{\bar{a}}_{\alpha }}\right\}\right].$$

One of the most troublesome problems of the interval and fuzzy arithmetic is the so-called excess width effect, i.e., the rapid growth of the widths of the resulting intervals with an increase in the number of arithmetic operations in the problems being solved. Despite the fact that the growth of interval widths in this case seems inevitable and corresponds to the general methodological provisions, in practice this phenomenon can lead to unacceptable results.

In this regard, the developed method of fuzzy calculation of the local criteria ranks was tested.

**Figure 4:**
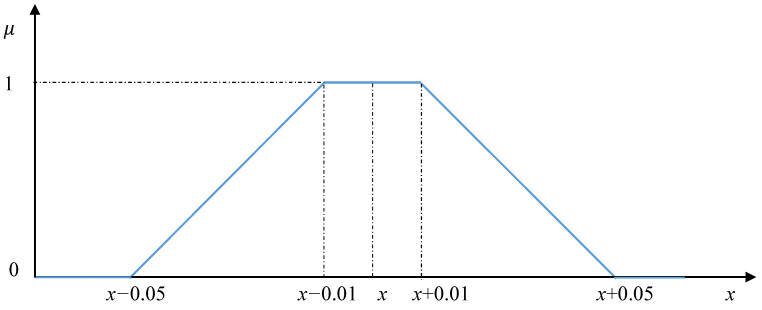
The fuzzy extension of the pairwise comparison matrix components.

The matrix of pairwise comparisons presented in Table 3 was used as a real-valued basis. Further, all elements of this matrix were replaced with fuzzy numbers. The average values *x* of fuzzy numbers were equal to the values of the elements of the original matrix. The fuzzy values in all cells of the matrix had the same maximum width equal to 0.1 (Figure 4).

The results of calculation of the final fuzzy local criterion weights in comparison with the calculated real-valued weights are presented in Table 6.

As can be seen from the Table 6, the maximal widths ($x_{4}-x_{1}$) of the resulting fuzzy weights in all cases are significantly less than the maximal widths of the original fuzzy values (see Figure 4), which are elements of the fuzzy matrix of pairwise comparison. It is clear that the index IR is also implemented in the fuzzy form.

The results of testing the developed method for calculating fuzzy weights indicate that there is no effect of intensive growth in the width of the final fuzzy values. There is a completely opposite effect of narrowing the widths of intervals, which at first glance, contradicts the original methodological principles of operating with uncertainties. However, a more detailed analysis of the situation shows that in cases where the operations of exponentiation and division lead to a decrease in the usual crisp values, applying them to fuzzy values built on the basis of the original real-valued ones reduces the widths of the results.

You can see that the very semantics of the word “division” implicitly implies some kind of splitting, reduction by obtaining smaller parts of a whole, etc. It is interesting to note that the subtraction operation in the conventional interval and fuzzy arithmetic always leads to an increase in the widths of the final values, which, however, is not surprising if we analyze the situation of subtracting a negative value from a positive one.

Summarizing, we can say that usually obtaining local criterion weights is so uncertain a problem that the use of more complex methods (see, e.g., [58,59]) than that based on the direct fuzzy extension of (see (3)) seems to be methodologically unjustifiable.

Additionally, taking into account that the practical need for fuzzy weights appears very rarely, we did not include the fuzzy extended approximate method (3) in our set of selected mathematical tools for the solution of the fuzzy MCDM problems and use it separately whenever such a need really arises.

Therefore, as a basic tool for calculating real-valued local criterion weights, we use the method (2) based on the nonlinear optimization task.

Like any well-known numerical method, it has a number of disadvantages identified in the course of its intensive applications.

Here we consider the most important and discussed one—the rank-reversal problem. This problem is that when a new criterion or new alternative is added to the MCDM problem, the ranking of the previous old group of criteria may be violated. Currently there is not a consensus concerned with the positive vs. negative treatment of this phenomenon, as some scholars based on surely reasonable arguments consider it as natural and desirable one, while others completely reject it (see discussion in [61]).

There are some approaches proposed in the literature that allow a decision maker to complete avoid ly the rank reversal effect [62,63]. Nevertheless, these approaches are not well matured yet, and currently all their inevitable shortcomings and limitations are not known.

On the other hand, based on our—but not only our—experience, we can say that when using the model (2), we tackle it with the rank reversal effect very rarely and often when it may be explained in a reasonable way. Therefore, here we will use a reliable, proven by practice method for calculating the local criterion weights based on the nonlinear optimization task (2).

### 2.3. Local Criteria Convolution Problem

Once all local criteria and their weights are established, to obtain the generalized numerical estimation of an alternative they should be convoluted (aggregated) into the general criterion using an appropriate approach.

There is a general perception that it is impossible to formulate the best local criteria convolution (aggregation) method for all possible cases. It is stated in [64] that the choice of an appropriate convolution is the problem dependent on the context of the issue. However, there are general theoretical and practical reasons to assess the relative reliability of the most commonly used local criteria convolution methods (see [41,43]).

To simplify the analysis, let us consider a two-criterion situation. Let X and Y be the local criteria with their corresponding membership functions $\mu_{X}$ and $\mu_{Y}$, and the weights $w_{X}$ and $w_{Y}$. Then, for each a∈A, where A is the set in the space of alternatives, the values of functions $\mu_{X}\left(a\right)$ and $\mu_{Y}\left(a\right)$ can be calculated. Of course, *a* is not a variable in the usual sense. Rather, it is a label assigned to the corresponding alternative.

Among the many possible convolution methods, the most commonly used are the following three, which can also serve as a basis for constructing more complex convolutions:

- Additive convolution (weighted sum):
  (5) $$C_{add}=w_{X}\mu_{X}+w_{Y}\mu_{Y};$$
- multiplicative convolution (Harrington [46], 1965)
  (6) $$C_{prod}=\mu_{X}^{w_{X}}*\mu_{Y}^{w_{Y}};$$
- min convolution (maximal pessimism) (Yager [65], 1979)
  (7) $$C_{min}=min\left(\mu_{X}^{w_{X}},\mu_{Y}^{w_{Y}}\right).$$

The extension of the above definitions to the case of more criteria is obvious and therefore not presented.

To simplify the analysis, let us consider the situations of the continuous sets of alternatives. For this case, we have proved in [45] the theorems that are valid for both decision-making and multi-criteria optimization problems. These theorems are graphically illustrated in our papers [41,43].

It is shown in [41,43] that the weighted sum convolution (5) provides counter-intuitive illogical results in the Pareto region. Slightly better results may be obtained using the multiplicative convolution (6). The min type convolution has no such drawbacks and should be recognized as the best one. This is the most cautious approach, as in the optimization task we are seek the best solution in the most pessimistic scenario.

To clarify the features of the convolutions methods (5)–(7) consider the following example.

In many countries, the degree of air pollution *D* is assessed as follows:

(8) $$D=\frac{1}{n}\sum_{i=1}^{n}\alpha_{i}\frac{C_{i}}{C_{i(per)}},\;\sum_{i=1}^{n}\alpha_{i}=n,$$

where $C_{i}$ is the current concentration of the chemical harmful to health; $C_{i(per)}$ is the maximum permissible concentration of this chemical according to the standard. $\alpha_{i}$ is the degree of harmfulness of a given chemical.

Unacceptable air pollution conditions occurs when D>1, under acceptable conditions 0≤D≤1.

Let us say there is an ice cream factory in the center of the town. It causes almost no air pollution (all). However, in the industrial refrigerator, the chemical NH_3_ is used as a refrigerant. Suppose there was a refrigerator failure and the concentration of NH_3_ suddenly increases and is 15 times greater than $C_{{\mathrm{NH}}_{3}\left(per\right)}$. For simplicity of the analysis, let us assume that $\alpha_{{\mathrm{NH}}_{3}}$ = 1. Then from the above expression (8), given that *n* = 60, we get *D* = 0.4. We can see that formally, based on (8), the ecological situation in the vicinity of the ice cream factory is still better than in the vicinity of the chemical plant. However, there is no one who can judge this (everyone in the vicinity of the ice cream factory died because NH_3_ is a deadly, potent poison).

As follows from the analysis presented in [66], today the additive convolution is not used at all in ecological research precisely as the result of the problems detected above.

In other applications, there are situations less spectacular that are also evidence of the additive convolution’s shortcomings.

It can be said that it was precisely this kind of circumstance that caused the classical theories of decision-making (e.g., utility theory) to be treated today rather as branches of pure mathematics.

On the other hand, let us deal with stock trading. Suppose at some time we analyze the ten indicators used and nine of them are greater than zero, generating the buy signal to varying degrees, whereas the remaining indicator is equal to zero, suggesting to refrain from buying.

Obviously, the convolutions (6) and (7) will be equal to zero and therefore we should reject buying.

On the other hand, in the case under consideration, we have nearly ten times more arguments in favor of buying than in favor of refusing it and should (of course with some risk) buy the stocks. It is easy to see that this decision is supported by the value of type (5) general criterion.

It is worthy to note that the important property of the additive convolution (5) is that the nearly zero values of some criteria may be completely re-compensated by the large values of the other ones.

Based on the above analysis, we can say that this property may be treated as positive or negative dependent on the MCDM problem at hand.

In many practically important cases, we do not have reliable information for a priori selection of the convolution type. Hence, the problem of the generalization of the convolution modes and finding an appropriate consensus solutions arises. The corresponding technique developed to solve these problems will be presented in Section 2.5.

### 2.4. Hierarchical Sets of Local Criteria

The hierarchical structure of local criteria often occurs even in the relatively simple MCDM tasks. The tremendous number of the publications devoted to the use of the analytic hierarchy process (AHP) [15] with its modifications for the solution of the hierarchical MCDM problems causes the dominance of this very controversial method over other approaches that deserve no less attention.

We avoid here the detailed description of the AHP since it may be found in the numerous textbooks, and therefore focus on its problems and the presentation of our method which is free of these problems.

There were many weaknesses and shortcomings of the AHP revealed during its intensive applications to the solution of the MCDM problems. Here we present only some of them which seem to be more important.

The most debatable problem is the rank reversal effect (described above in Section 2.2) that likely occurs, e.g., when nearly the same new alternative as an existing one is included to the set of analyzed alternatives.

It was proved in [67] that this effect does not appear if the multiplicative modification of the AHP is used. As it is shown in [68], the core of the rank reversal problem in the AHP is the local criterion weights interpretation.

Despite of this, the AHP with some modifications is accepted by many scholars as the most reliable MCDM method. Generally the AHP may be treated as a fully additive convolution method.

The compensatory property of this convolution (see Section 2.2) may be treated as a positive or negative one in different situations, but most researchers consider it as a non-desirable one as it may provide the loss of important information.

One of the disadvantages of AHP is that the number of pairwise comparisons needed to implement the method often happens to be very large (n(n−1)/2), so the AHP seems to be an expensive task. Another important witness to the AHP method is the artificial limitation based on the use of a restricted the 9-point scale. The AHP method cannot work in the situations when, e.g., an alternative *A* is 15 times more important than alternative *C* due to the scale’s restrictions. To simplify the basic AHP method and to reduce its shortcomings, in [69] proposed a modified procedure using a 2-point-scale. In this approach, the decision maker only specifies whether a criterion is more or less important or equally important than another compared one.

In [70], a negative feature of the AHP that may be a stronger shortcoming of the method than the rank reversal affect was discussed. It is based on the observation that the addition of indifferent criteria (for which all alternatives perform equally) provides a great alteration of the aggregated alternatives evaluations with undesired results. Therefore, in the relatively complex hierarchies (with more than three levels), the rank reversal effect may appear. Since in almost all applications of the AHP, the set of criteria is not fixed a priory, but is variable and is built based on the reasons of appropriateness and simplicity, likely almost all applications of the AHP are potentially flawed.

The known approaches to the improvement of the AHP do not, however, violate its basic structure. This structure imposes a joint reassessment of all alternatives when adding each new one.

According to the opinions of many researchers, it is this feature of the AHP that is the main cause of the rank reversal effect. Nevertheless, it is clear that if we will accomplish the assessments of alternatives only separately, the causes of the appearance of this effect disappear. Therefore, in our work [43], we proposed an appropriate and simple enough scheme of the local criteria hierarchy representation.

**Figure 5:**
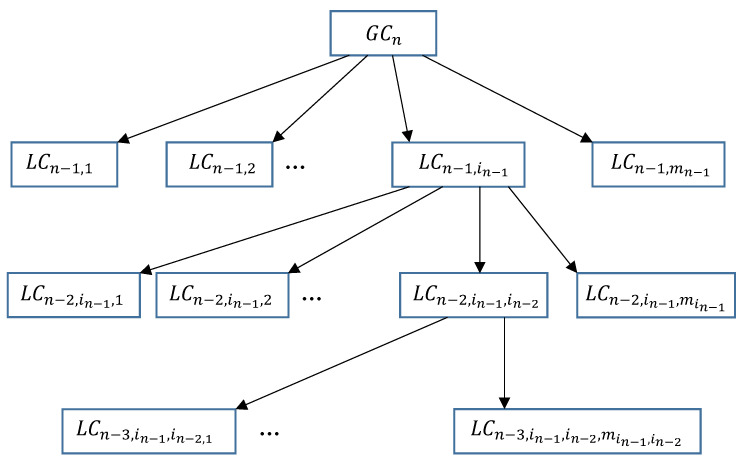
General scheme of building a hierarchical structure of local criteria.

The developed method allows us to build in a natural way the multi-leaf and hierarchical structures, the scheme of which is shown in Figure 5.

Each higher-order criterion is constructed on the basis of lower-order local criteria being aggregated or by a combination of convolution methods. A general mathematical expression for the calculation of criteria at the intermediate levels of the hierarchy can be presented in the following form:

(9) $$LC_{n-1,i_{n-1}}=F_{n-1,i_{n-1}}\left(LC_{n-1,i_{n-1},1},\ldots ,LC_{n-2,i_{n-1},m_{i_{n-1}}},w_{n-1,i_{n-1},1},\ldots ,w_{n-2,i_{n-1},m_{i_{n-1}}}\right),$$

where $F_{n-1,i_{n-1}}$ is the operator of local criteria convolution; $LC_{n-2,i_{n-1},m_{i_{n-1}}}$ are the intermediate local criteria at the corresponding levels of the hierarchy and $w_{n-1,i_{n-1},1}$ are the relative weights corresponding to them.

As follows from the way the structure is built, $LC_{n-1,i_{n-1}}$ always take values between 0 and 1. At the lowest level of the hierarchy, the direct membership functions of the primary local criteria determined by the basic quality parameters are used:

(10) $$LC_{1,i_{n-1},i_{n-2},\ldots ,i_{2}}=F_{1,i_{n-1},i_{n-2},\ldots ,i_{2}}\left(\left\{\mu_{j}\right\},\left\{w_{j}\right\}\right).$$

Figure 5 shows a general way of creating the multiple criteria hierarchical structures within the developed method.

The building a hierarchical system using the developed method consists of the following stages:

- Creating a “tree structure” for the problem under consideration;
- Mathematical formalization of the membership function at the lowest level;
- Filling in the matrix of pairwise local criteria comparisons at the lowest level;
- convolution of local criteria at the lowest level;
- Filling in the matrix of pairwise comparisons of local criteria at intermediate levels of the hierarchy and assessing their relative weights;
- Computing the global assessment of aggregated criteria by convoluting them from bottom to top.

The value of generalized criterion $LC_{n}$ is the final evaluation of the considered alternative obtained independent on the other alternatives at hand.

The developed method of multiple criteria hierarchical evaluation of alternatives can also be interpreted in terms of fuzzy logic.

**Figure 6:**
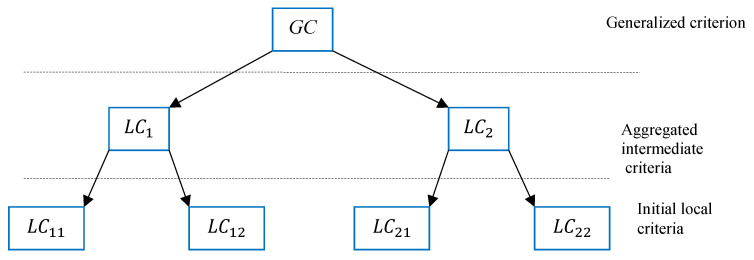
Two-level MCDM problem.

Consider an example of two-level hierarchy presented in Figure 6.

Using fuzzy logic terminology, the decision process based on the calculation of the value of the generalized criterion GC, can be represented as follows:

(11) $$\begin{array}{c} \mathrm{if}\;LC_{1}\;is\;C_{1}\;and\;LC_{2}\;is\;C_{2}\;\mathrm{then}\;GC=AGG\left(w_{1},w_{2},LC_{1},LC_{2}\right)\\ \;e.g.:\;AGG=min\left\{LC_{1}^{w_{1}},LC_{2}^{w_{2}}\right\};\;AGG=w_{1}LC_{1}+w_{2}LC_{2};\;AGG=LC_{1}^{w_{1}}*LC_{2}^{w_{2}}\\ \mathrm{if}\;x_{11}\;is\;A_{11}\;and\;x_{12}\;is\;A_{12}\;\mathrm{then}\;LC_{1}=AGG\left(\mu_{11}\left(x_{11}\right),\mu_{12}\left(x_{12}\right),w_{11},w_{12}\right)\\ \mathrm{if}\;x_{21}\;is\;A_{21}\;and\;x_{22}\;is\;A_{22}\;\mathrm{then}\;LC_{2}=AGG\left(\mu_{21}\left(x_{21}\right),\mu_{22}\left(x_{22}\right),w_{21},w_{22}\right)\\ \;e.g.:\;AGG=min\left\{\mu_{21}^{w_{21}}\left(x_{21}\right),\mu_{22}^{w_{22}}\left(x_{22}\right)\right\};\\ \;\;\;\;AGG=w_{21}\mu_{21}\left(x_{21}\right)+w_{22}\mu_{22}\left(x_{22}\right);\\ \;\;\;\;AGG=\mu_{21}^{w_{21}}\left(x_{21}\right)*\mu_{22}^{w_{22}}\left(x_{22}\right), \end{array}$$

where $w_{1}$, $w_{2}$, $w_{11}$, $w_{12}$, $w_{21}$ and $w_{22}$ are the weights of the corresponding local criteria.

In the above expressions (11), the conditions of type $x_{11}$ is $A_{11}$ can be interpreted linguistically as “$x_{11}$ meets the conditions of the criterion $A_{11}$”. In this case, the value $\mu_{11}\left(x_{11}\right)$ is the degree of fulfillment of these conditions or the “degree of satisfaction” of the criterion $A_{11}$. The obtained value of $LC_{1}$ is the already calculated degree of satisfaction of the intermediate local criterion $LC_{1}$ with respect to the premises of the local criteria $A_{11}$ and $A_{12}$. Based on these intermediate calculations, finally the general evaluation GC is obtained.

If the parameters $x_{11}$ and $x_{12}$ are fuzzy then the values of the local criterion $LC_{1}$ will be fuzzy as well.

In terms of decision-making reliability, the defuzzification operation is not only unnecessary, but even not desired, especially at intermediate levels of the hierarchy. By avoiding defuzzification at the highest level of the hierarchy, we obtain estimates of alternatives $GC(al_{i})$ in the form of fuzzy values ($al_{i}$ is the competing alternative, i=1,…,n). The best alternative should maximize the fuzzy value of $GC(al_{i})$. To find this alternative, we use a probabilistic approach to compare fuzzy numbers (see our paper [40]).

Of course, the aggregation operator AGG is constructed in such a way that the maximum degree of satisfaction of the criterion $C_{1}$ corresponds to the value $C_{1}$ = 1 and the minimum degree of satisfaction of this criterion is zero value.

An important difference in our approach to the solution of the hierarchical fuzzy MCDM problems from the commonly used AHP is that in contrast to our approach, the AHP does not allow us to use the fuzzy representations of the local criteria described in the Section 2.1. Sometimes this is exposed as an advantage of the AHP providing an opportunity to solve the MCDM problems even in the cases when there is no quantitative information of local criteria. Nevertheless, in the most of cases we met in our practice, the membership functions representing fuzzy local criteria were built based directly on the commonly used information, e.g., technological instructions in the industry (see Section 2.1 for the simple method of such fuzzy criteria building) or diagnostic protocols in the medicine. Therefore, the artificial exclusion of this kind of important quantitative information (fuzzy criteria) only in order to be able to use AHP looks very unjustified. Hence, we can say that the direct use of fuzzy criteria in our method is its most important advantage compared to the AHP.

As there are many possible convolution (aggregation) methods proposed in the literature, the problem to develop an appropriate method for the generalization of convolution modes that may provide a consensus solution of the MCDM problems, naturally arises. The developed method of the generalization of convolution modes based on the synthesis of the type 2 and level 2 fuzzy sets is presented in the next subsection.

It is worth emphasizing that only independent local criteria are considered in the framework of the developed approach. However, we have observed that in practice, for example, when making a decision to buy or sell securities on the basis of the market’s technical analysis data, the relative importance of the criteria depends on the degree of execution. At the same time, experienced traders formulate this problem verbally, for example, as:

(12) $$\begin{array}{c} \mathrm{if}\;\mu_{11}\left(x_{11}\right)\;is\;medium\;and\;\mu_{12}\left(x_{12}\right)\;is\;low\;\mathrm{then}\;w_{11}\approx w_{12},\\ \mathrm{if}\;\mu_{11}\left(x_{11}\right)\;is\;large\;and\;\mu_{12}\left(x_{12}\right)\;is\;low\;\mathrm{then}\;w_{11}>>w_{12}. \end{array}$$

It follows that practice imposes the use of the synthesis of decision-making and fuzzy logic methods, but this is outside the scope of this paper.

### 2.5. Generalization of the Local Criteria Convolutions

In practice, it is usually impossible to establish a priori the best in the considered situation method for convolution of local criteria. Besides such methods may generate substantially different rankings of alternatives. Therefore the problem of formal mathematical generalization of convolution methods allowing us to obtain the compromise solutions of the MCDM tasks becomes very urgent [71].

So in [72], for this purpose, the tools of the possibility theory are used. In [73], the so-called weighted averaging operation is proposed. The method based on the Yager’s t-norm is developed in [74]. The method of hierarchical aggregation is developed in [75,76].

Currently, the generalization of convolution modes using the so-called γ–operator is widely used [64,77]:

(13) $$\beta ={\left(\prod_{i}m_{i}\right)}^{1-\gamma }{\left(1-\prod_{i}\left(1-m_{i}\right)\right)}^{\gamma },\;i=1,2,\ldots ,n;\;0\leq \gamma \leq 1,$$

where $m_{i}$ is a membership function of the local criterion.

It is clear that this expression is based only on the multiplicative convolution. The more general approach based on minimum, maximum and additive convolutions was proposed in [78]:

(14) $$\beta_{mixt}=\gamma \min_{i}\left(m_{i}\right)+\left(1-\gamma \right)\left(\sum_{i}m_{}i\right)/n.$$

The problems with this approach are that the local criteria are not weighted and the additional information concerned with the choice of an appropriate value of parameter γ is needed.

The expressions (14) were used in [79] for the solution of multi-level decision-making problems. As the main problem, the lack of strict rules for choosing the parameter γ was noted. In [80], a method is proposed that formalizes to a certain extent the choice of γ, but it requires to get a significant amount of additional quantitative information from the experts. It should be noted that in (14) the local criteria are assumed to be of equal importance. It is clear that their ranking using, for example, the method of pairwise comparisons, is a more complex and responsible task than the choice of the parameter γ. In addition, the considered approaches do not allow us to aggregate simultaneously all three main types of the local criteria convolutions based on the min operator, additive and multiplicative operators.

The approaches discussed above do not allow us for the simultaneous aggregation of all relevant criteria using combinations of min, additive and multiplicative operators. Of course, using these operators for the generalization of convolution methods is not a solution to the problem because the resulting generalizations need further generalizations, etc., indefinitely.

Therefore, a method for the generalization of convolution modes based on the synthesis of type 2 and level 2 fuzzy sets has been developed in our papers [41,43] to avoid the use of the original convolution operators for their further generalization.

Then first, we briefly present the basic definitions of type 2 and level 2 fuzzy sets only in the extent needed for the subsequent analysis.

#### 2.5.1. Type 2 Fuzzy Sets

The concept of type 2 fuzzy sets were first introduced by L.A. Zadeh [81] in relation to the linguistic variable presentation and further developed in [82,83,84].

Let *X* be a fuzzy set of type 2 on *A*. Then for every a∈A there is a fuzzy set on *B* and there is a membership function (for a discrete set):

(15) $$\mu_{X}\left(a\right)=\left\{\frac{F_{a}\left(b_{i}\right)}{b_{i}}\right\},\;i=1,\ldots ,n,$$

which is a fuzzy set on *B* characterized by the membership function $F_{a}\left(b\right)$. The complement of the type 2 fuzzy set under consideration can be represented by:

(16) $$\mu_{\bar{X}}\left(a\right)=\left\{\frac{F_{a}\left(b_{i}\right)}{1-b_{i}}\right\},\;i=1,\ldots ,n.$$

If *X* and *Y* are fuzzy sets of type 2 with the membership functions

(17) $$\mu_{X}\left(a\right)=\left\{\frac{F_{a}\left(b_{i}\right)}{b_{i}}\right\},\;\mu_{Y}\left(a\right)=\left\{\frac{d_{a}\left(e_{j}\right)}{e_{j}}\right\}\;i=1,\ldots ,n,\;j=1,\ldots ,m,$$

then the membership function of the set G=X∪Y takes the form

(18) $$\mu_{G}\left(a\right)=\left\{\frac{F_{a}\left(b_{i}\right)\wedge d_{a}\left(e_{j}\right)}{\left(b_{i}\vee e_{j}\right)}\right\},\;i=1,\ldots ,n,\;j=1,\ldots ,m.$$

Similarly for the intersection R=X∩Y we have

(19) $$\mu_{R}\left(a\right)=\left\{\frac{F_{a}\left(b_{i}\right)\wedge d_{a}\left(e_{j}\right)}{\left(b_{i}\wedge e_{j}\right)}\right\},\;i=1,\ldots ,n,\;j=1,\ldots ,m.$$

For simplicity and some concretization, we do not use here a generalized representation of operations with the fuzzy sets presented by t-norms and s-norms.

#### 2.5.2. Level 2 Fuzzy Sets

For the first time, the level 2 fuzzy sets were introduced by L.A. Zadeh [85] and then were studied and extended in [86,87]. According to L.A. Zadeh [88], the level 2 fuzzy set is the fuzzy set based on the support consist of usual fuzzy sets. If the elements a∈A are fuzzy subsets of some other set *E*, then in a discretized case, the fuzzy set *X* of level 2 can be represented as follows:

(20) $$\begin{array}{c} X=\left\{\frac{\mu_{X}\left(a_{i}\right)}{a_{i}}\right\},\;a_{i}=\left\{\frac{q_{i}\left(e_{j}\right)}{e_{j}}\right\},\\ X=\left\{\frac{\max_{i}\left[\mu_{X}\left(a_{i}\right)q_{i}\left(e_{j}\right)\right]}{e_{j}}\right\},\;j=1,\ldots ,m. \end{array}$$

From the last expression it follows that the degree of membership of $e_{j}$ to *X* is

(21) $$\mu_{X}\left(e_{j}\right)=\max_{i}\left[\mu_{X}\left(a_{i}\right)q_{i}\left(e_{j}\right)\right],\;j=1,\ldots ,m.$$

We will use the mathematical tools of the type 2 and level 2 fuzzy sets described above to generalize the methods of local criteria convolution, aiming to obtain a some kind consensus solution of the MCDM problem at hand.

Let is have $K_{m}$ relevant methods of local criteria convolution and $M_{m}$ selected local criteria. Let then $\mu_{C_{i}}$, $i=1,\ldots ,K_{m}$ be the membership function reflecting the subjective opinions of decision makers concerned with the “proximity” of the particular *i*th convolution method $C_{i}$ to the some “ideal” convolution method satisfying all possible criteria and demands, which possible may not be even formulated explicitly at the verbal level.

Let us emphasize that $\mu (C_{i})$ can be represented by the experts at the linguistic level, and then $\mu (C_{i})$ can be formalized in the form of the fuzzy sets. Then this “ideal” convolution method can be represented as the type 2 fuzzy set:

(22) $$C_{id}=\left\{\frac{\mu (C_{i})}{C_{i}}\right\},\;i=1,\ldots ,K_{m},$$

where $C_{i}$ denotes the used convolution method, e.g., $C_{1}$ (5), $C_{2}$ (6) and $C_{3}$ (7).

In turn, each $C_{i}$ can be presented as the fuzzy set based on the usual set of alternatives considered. Then we have:

(23) $$C_{i}=\left\{\frac{C_{i}\left(e_{j}\right)}{e_{j}}\right\},\;j=1,\ldots ,M_{m},$$

where $e_{j}$ is the alternative, $C_{i}\left(e_{j}\right)$ is the degree of adequacy of the alternative $e_{j}$ with respect to the convolution of type $C_{i}$.

Substituting (23) into the expression (22), we obtain the mathematical construction that is simultaneously a fuzzy set of type 2 and level 2:

(24) $$C_{id}=\left\{\frac{\mu_{id}\left(e_{j}\right)}{e_{j}}\right\},\;j=1,\ldots ,M_{m},$$

where

(25) $$\mu_{id}\left(e_{j}\right)=\max_{i}\left(\mu \left(C_{i}\right)*C_{i}\left(e_{j}\right)\right),\;j=1,\ldots ,M_{m}.$$

Of course, the best alternative $e_{j}$ will be that providing the largest value of $\mu_{id}\left(e_{j}\right)$.

#### 2.5.3. Consider an Example

Suppose a set of four tender applications $TA_{i}$, i = 1 to 4, was evaluated. Let us assume that using the methods of the local criteria convolution, e.g., $C_{1}$, $C_{2}$ and $C_{3}$ of the same types as (5), (6) and (7) presented earlier, the assessments of the attractiveness of the competing $TA_{i}$ were made and the results are presented in Table 7.

It is not clear from the results obtained, what the tender application is the most attractive. Of course, intuitively we can suspect that $TA_{3}$ is better than the others because for this tender application the results of two convolutions $C_{1}$ and $C_{2}$ are better than the other. On the other hand, the convolution $C_{3}$ provides the greatest results for $TA_{2}$. It is seen that such an uncertain reasoning do not enough to make a final decision. Therefore, to obtain an unambiguous result, we will use the previously presented method for the generalization of convolution modes.

According to this method, to solve the problem of multiple criteria evaluation of the tender applications under consideration, we should first find the coefficients of relative reliability (weights) of the used convolution methods $C_{1}$, $C_{2}$ and $C_{3}$. Based on the analysis provided in [37], the following verbal assessments of the relative reliability of the considered methods for convolution can be accepted: $C_{3}$ is somewhat better than $C_{2}$, whereas and $C_{3}$ and $C_{2}$ are definitely better than $C_{1}$. Based on these verbal estimations, the corresponding pairwise comparisons matrix presented in Table 8 was obtained.

Based on the this matrix, we the coefficients of the relative reliability of the considered methods for convolution were calculated as follows: $\mu (C_{1})$ = 0.07, $\mu (C_{2})$ = 0.35, $\mu (C_{3})$ = 0.58.

Then the “ideal” generalized global convolution method can be represented as the fuzzy set defined on the set of considered convolution methods:

(26) $$C_{id}=\left\{\frac{\mu (C_{1})}{C_{1}},\frac{\mu (C_{2})}{C_{2}},\frac{\mu (C_{3})}{C_{3}}\right\}=\left\{\frac{0.07}{C_{1}},\frac{0.35}{C_{2}},\frac{0.58}{C_{3}}\right\}.$$

In turn, each of $C_{1}$, $C_{2}$ and $C_{3}$ can be represented as the fuzzy sets in the space of the considered tender applications:

(27) $$C_{i}=\left\{\frac{C_{i}\left(TA_{1}\right)}{TA_{1}},\frac{C_{i}\left(TA_{2}\right)}{TA_{2}},\frac{C_{i}(TA_{3}}{TA_{3}},\frac{C_{i}\left(TA_{4}\right)}{TA_{4}},\right\}\;i=1,..3,$$

where $C_{i}\left(TA_{j}\right)$ is the evaluation of the *j*th TA by the *i*th convolution method.

Taking into account (26) and (27), it can be concluded that $C_{id}$ is the fuzzy set of level 2. Hence, substituting (27) in (26) we get:

(28) $$C_{id}=\left\{\frac{\mu_{id}\left(TA_{1}\right)}{TA_{1}},\frac{\mu_{id}\left(TA_{2}\right)}{TA_{2}},\frac{\mu_{id}\left(TA_{3}\right)}{TA_{3}},\frac{\mu_{id}\left(TA_{2}\right)}{TA_{2}}\right\},$$

where

(29) $$\mu_{id}\left(TA_{j}\right)=\max_{i}\left(\mu \left(C_{i}\right)\cdot C_{i}\left(TA_{j}\right)\right)$$

is the final evaluation of the *j*th tender application $TA_{j}$ based on the generalization of all three considered types of convolution.

From the expression (28) it is clear that for the final choice of the best tender application based on the local criteria convolution and generalization of convolution modes, we should find the tender application characterized by the maximum value of $\mu_{id}$. Using the data obtained as the results of the local criteria convolution presented in Table 8 and the values of their relative reliability $\mu (C_{1})$ = 0.07, $\mu (C_{2})$ = 0.35, $\mu (C_{3})$ = 0.58, calculated from the expression (29), we obtain the results presented in Table 9.

As can be seen from the Table 9, the best alternative is the tender application 3. This conclusion confirms the previously provided (on an intuitive level) result of analysis of the local criteria convolutions (see Table 7). However, it should be borne in mind that when making decisions, we may encounter much more controversial situation when we will not be able to make such a qualitative analysis. The presented method for the generalization of convolution modes allows us to obtain the consensus solution of such complicated MCDM problems.

## 3. An Example of the Hierarchical Fuzzy MCDM Problem Solution (the “Cat Buying” Problem)

Let us consider the problem of cat shopping. This problem, seemingly trivial at first glance, may become not so simple if there is a pet shop big enough nearby.

Then our purpose is to provide decision-making support in problems of fuzzy multiple-criteria hierarchical choices of cats.

As to the solution of this problems, a corresponding application based on the mathematical tools presented in Section 2 was developed.

Let us consider the algorithm of this application step by step based on the example of buying a cat problem.

**Figure 7:**
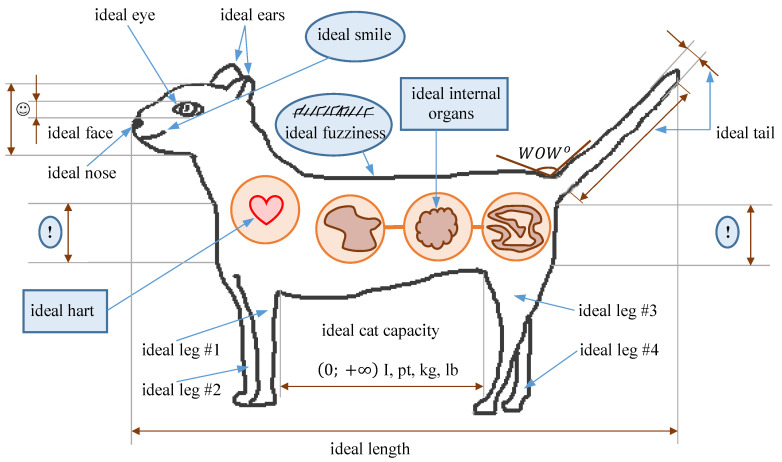
The possible local criteria of cat quality.

Figure 7 presents one of the possible hierarchies of the local criteria we may come up with when considering the problem.

Let us comment on it little bit more. For the sake of simplicity, limit the number of major parameters of cat’s quality to three: “fuzziness in cm”, “activity” and “weight in kg”.

Obviously, since when buying a cat we hope to get a friend, money does not matter.

**Figure 8:**
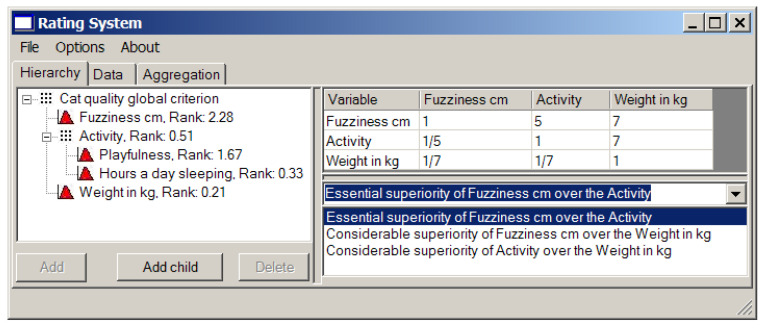
The local criterion weights (ranks) and the pairwise comparison matrix.

First obtain the weights of local criteria using the matrices of pairwise comparison (see Figure 8).

Generally, we like fuzzy animals, so “fuzziness in cm” is our top priority. The numbers 5 and 7 in the first row of the pairwise comparison matrix (the right window in Figure 8) mean that “fuzziness” is essentially more important for us than “activity” and considerably more important than “weight in kg”.

On the other hand, we would like to have a playful cat. Hence, “activity” gets number 7 against “weight in kg”.

**Figure 9:**
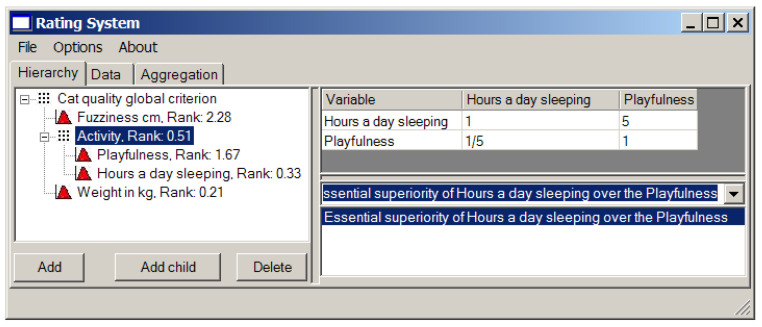
Playfulness against laziness.

“Activity” is quite a vague (fuzzy) notion, so we separate it onto two more parameters: overall playfulness of a cat on the scale from 0 (absolutely inert creature) to 10 (twister with a tail), and, as a measure of laziness, the number of hours per day it spends sleeping. We prefer passive or over-active cats to the ones that are never awake (perhaps it is dead), so “hours a day sleeping” gets 5 against “playfulness” (see Figure 9). It is easy to see that in this case we deal with the hierarchical structure of local criteria.

The resulting ranks of all local criteria are presented in the left window in Figure 9.

Let us formalize the local criteria, representing them by the corresponding membership functions.

**Figure 10:**
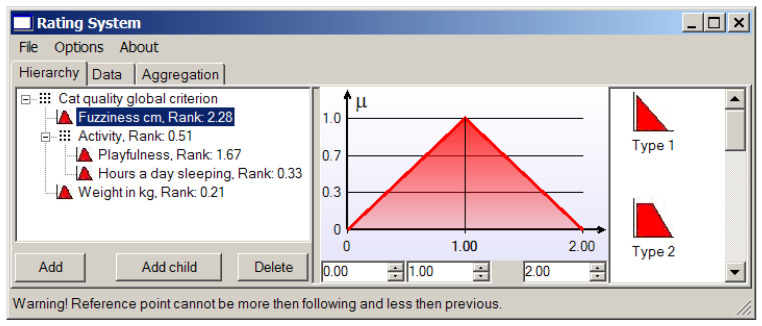
The local criterion based on the fur length.

Suppose we consider a cat’s fur to be perfect when its length is 1 cm, and we are not ready to deal with heaps of lint, so a cat with the two-centimeter length fur will do. The corresponding membership function for the criterion based on the fur length is shown in Figure 10.

We can see that the provided application allows us to choose first the type of membership function among those presented in Figure 3 and then establish needs in the considered case reference values (points) characterizing the concrete triangular or trapezoidal local criterion.

**Figure 11:**
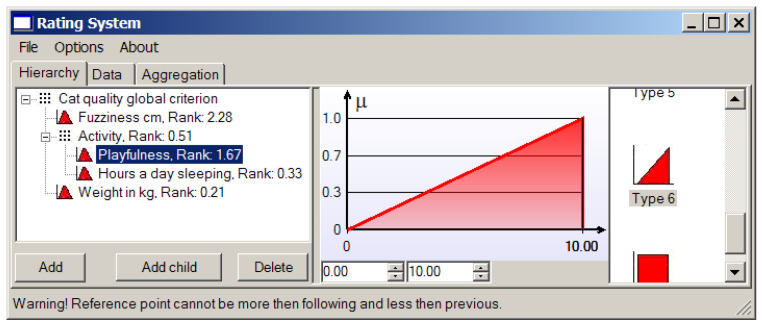
The membership function of the “playfulness” criterion.

Usually we love playful cats, so “twister with a tail” is our dream. The membership function representing the criterion based on this parameter is shown in Figure 11.

**Figure 12:**
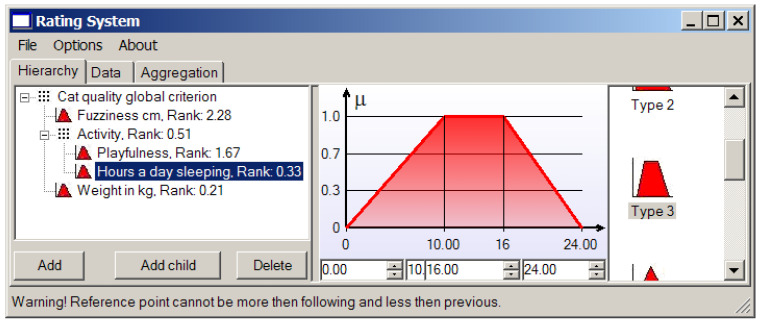
The membership function of the “laziness” (“hours a day sleeping”) criterion.

A cat sleeping more than 16 h sounds quite boring, and while remembering our fondness for playful cats, it is not wise to wish for a cat that sleeps less than 10 h a day (Figure 12).

**Figure 13:**
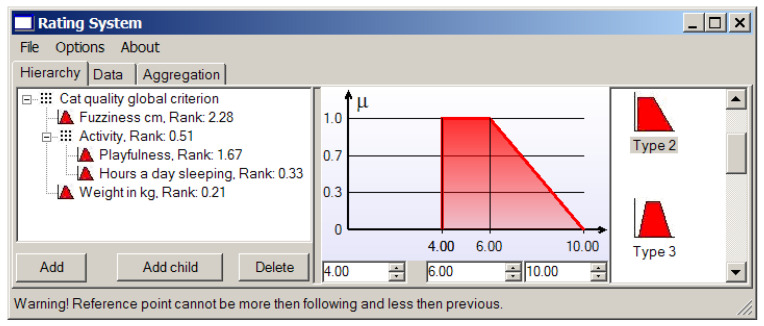
The membership function of “weight in kg” criterion.

We think a healthy cat should not weigh less than 4 kg, and it may be not so good of an idea to have a very active cat weighting more than 6 kg (Figure 13). Something more than 10 kg is becoming plainly dangerous.

**Figure 14:**
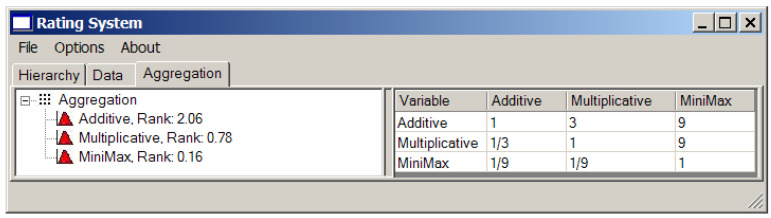
Ranking of the convoluting modes.

Since different methods for convolution of local criteria may be used, to obtain the compromise based on their generalization according the approach presented in Section 3, their relative importance should be established. In our case, we will use three types of convolution and the matrix of their pairwise comparison. The results are presented in Figure 14.

It is possible to change the relative ranks of convolution methods in the generalized criterion using the third tab “Aggregation” in the left window of application (see Figure 14).

**Figure 15:**
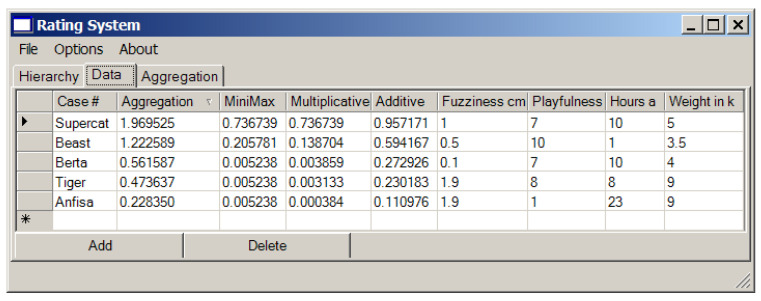
The results of the evaluation of the cats.

Now we are ready to go shopping. Suppose in the pet shop we can choose among the following cats: “Supercat”, “Beast”, “Berta”, “Tiger” and “Anfisa” which belong to different races. The parameters of these cats and the resulting values of convoluted criteria obtained using different convolutions modes are presented in Figure 15, where the results of their generalization are shown as well (see tab “Aggregation”).

We can see that in the considered example the compromised solution should be the choice of “Supercat”.

The presented application can be applied not only to help us choose a cat, but for the solutions of a considerably broad range of hierarchical fuzzy MCDM problems. The above presentation was aimed to convince the readers that building a relatively simple but useful and complete application supporting the solutions of the hierarchical fuzzy MCDM problems is not so difficult a task. We hope this aim will be achieved.

## 4. Conclusions

This paper is based on the experience accumulated by the authors during nearly forty years of practice focused on the development and implementation of the systems for the solution of the MCDM problems in different firms and institutions in Russia, Belarus and Poland. This experience was obtained in various branches of human activity, e.g., in technology, economics, ecology and medicine, under different types of uncertainties that inevitably accompany the MCDM problems.

By focusing on the practical aspects of the MCDM, the authors did not intend to provide the detailed descriptions of all known methods and presented only such mathematical tools and methodologies that were most often applied in their practice, while taking into account the types of uncertainty met in the concrete practical cases. That is why the paper may be considered as a practice-oriented illustrative review of the selected application-focused mathematical tools and methodologies most often used in the solution of the MCDM.

Hence, this paper is directly addressed to the practitioners who meet in their practice hierarchical fuzzy MCDM problems.

Based on the authors’ experience, it was found that in the majority of practical MCDM problems, we have insufficient information to use more complicated types of uncertainty than the usual fuzzy one. Often this does not mean that such complex uncertainties do not exist objectively; the decision makers and the experts involved just do not always have the necessary knowledge and skills to identify and formalize them.

Of course, in some relatively rare cases, the use and processing of more sophisticated forms of uncertainty is needed and justified. It was shown that the authors considerably contributed to the solution of such problems.

Many of the mathematical tools selected by the authors used in MCDM support are already available, but in this paper they are considered from the point of view of their synthesis into some general, but practical and not so complicated methodology for the solution of the hierarchical MCDM problems in the fuzzy setting. As an illustration of each methodology’s practical applicability, a complete user-friendly computer implementation of the selected mathematical tools is presented with the use of the simple “buying a cat” problem, which, however, has all the attributes of the hierarchical fuzzy MCDM task.

In Section 1, we have noted that many of new theories such as the L-fuzzy sets, the neutrosophic fuzzy sets, the Pythagorean fuzzy sets, the cubic fuzzy sets and so on, undoubtedly may be treated as innovative ones. However, now they are not mature enough and their practical applicability is not clear. Therefore, the preferred direction of our future work will be a deep analysis of these theories not only regarding their mathematical justifications, but mainly based on their applicability for the solutions of MCDM problems under these innovative types of uncertainty.

## Figures and Tables

**Table 1:** The linguistic estimates of relative importance.

Verbal Estimates	Corresponding Numbers
The two criteria are equivalent	1
Moderate superiority of the first parameter over the second	3
Moderate superiority of the first parameter over the second	5
Significant superiority of the first parameter over the second	7
Extremely strong superiority of the first parameter over the second	9
Intermediate estimates	2, 4, 6, 9

**Table 2:** An example of a pairwise comparison matrix.

	X	Y	Z
X	1	3	7
Y	1/3	1	3
Z	1/7	1/3	1

**Table 3:** The private house evaluation local criteria pairwise comparison matrix.

The Parameters of House Quality	The House Sizes	Convenience of Bus Routes	Surroundings	Age of the House	Yard	Modern Equipment	General Condition	Financial Condition
The house sizes	1	5	3	7	6	6	1/3	1/4
Convenience of bus routes	1/5	1	1/3	5	3	3	1/5	1/7
Surroundings	1/3	3	1	6	3	4	6	1/5
Age of the house	1/7	1/5	1/6	1	1/3	1/4	1/7	1/8
Yard	1/6	1/3	1/3	3	1	1/2	1/5	1/6
Modern equipment	1/6	1/3	1/4	4	2	1	1/5	1/6
General condition	3	5	1/6	7	5	5	1	1/2
Financial condition	4	7	5	8	6	6	2	1

**Table 4:** The random inconsistency values.

Matrix Size	1	2	3	4	5	6	7	8	9	10
Random consistency	0	0	0.58	0.90	1.12	1.24	1.32	1.41	1.45	1.49

**Table 5:** Ranks and inconsistency indexes in the house selection problem.

Local Criteria	Ranks	Ranks
of House Quality	(Approximate Method (3))	(Optimization Task (2))
The house sizes	0.173	0.137
Convenience of bus routes	0.054	0.054
Surroundings	0.188	0.121
Age of the house	0.018	0.030
Convenience of bus routes	0.031	0.046
Modern equipment	0.036	0.046
General condition	0.167	0.089
Financial condition	0.333	0.475
	$e_{max}$ = 9.669	$e_{max}$ = 9.387
	I = 0.238	I = 0.198
	IR = 0.169	IR = 0.14

**Table 6:** The calculated fuzzy weights and inconsistency index—inconsistency ratio (IR).

Number of Criterium	Real-Valued Weights	The Reference Points of the Fuzzy Values			
Corresponding to Fuzzy Weights			
$x_{1}$	$x_{2}$	$x_{3}$	$x_{4}$
1	0.173	0.155	0.171	0.179	0.197
2	0.054	0.050	0.060	0.065	0.076
3	0.188	0.131	0.145	0.152	0.169
4	0.018	0.013	0.018	0.020	0.026
5	0.031	0.028	0.034	0.037	0.044
6	0.036	0.033	0.040	0.044	0.052
7	0.167	0.147	0.163	0.171	0.189
8	0.333	0.325	0.344	0.355	0.380
OC	0.189	−0.006	0.148	0.230	0.411

**Table 7:** The results of the tender applications assessment.

	$C_{1}$	$C_{2}$	$C_{3}$
$TA_{1}$	0.28	0.03	0.37
$TA_{2}$	0.07	0.02	0.92
$TA_{3}$	0.599	0.240	0.85
$TA_{4}$	0.444	0.08	0.60

**Table 8:** The matrix of the pairwise comparison of convolution modes.

	$C_{1}$	$C_{2}$	$C_{3}$
$C_{1}$	1	1/8	1/8
$C_{2}$	8	1	1/3
$C_{3}$	8	3	1

**Table 9:** The final evaluations of the tender applications.

	$\mu_{\mathrm{opt}}$
$TA_{1}$	0.190
$TA_{2}$	0.064
$TA_{3}$	0.430
$TA_{4}$	0.287
